# Prosthetic Joint Infection due to *Histoplasma capsulatum* in a Patient from Trinidad: Workup, Pathology, and Treatment

**DOI:** 10.1155/2022/8998996

**Published:** 2022-08-05

**Authors:** James Satalich, Julie Reznicek, Alexandra Bryson, Prayag Pershad, Nicholas Hooper, Jibanananda Satpathy

**Affiliations:** ^1^Department of Orthopaedic Surgery, Virginia Commonwealth University Hospital System, Richmond, Virginia 23219, USA; ^2^Department of Infectious Disease, Virginia Commonwealth University Hospital System, Richmond, Virginia 23219, USA; ^3^Medical School, Virginia Commonwealth University Hospital System, Richmond, Virginia 23219, USA

## Abstract

*Histoplasma capsulatum* is a rarely reported cause of prosthetic joint infections. This current case report is of a patient from Trinidad, with a history of a right total knee replacement (TKR), who underwent a successful two-stage revision due to a *Histoplasmosis capsulatum* periprosthetic joint infection (PJI). This case report offers a unique treatment plan to successfully treat *Histoplasmosis capsulatum* periprosthetic joint infections and emphasizes the importance of obtaining an accurate travel history.

## 1. Introduction

Histoplasmosis is the most common endemic mycosis worldwide, with higher prevalence in tropical to temperate regions [1]. After inhalation of *H. capsulatum* spores from soil contaminated with bird or bat droppings, patients can develop pulmonary and extrapulmonary manifestations or remain asymptomatic [2]. Despite an increase in endemic mycoses worldwide, musculoskeletal manifestations remain quite uncommon [3]. There have been limited documented cases of prosthetic joint involvement in the United States [4–7]. At a large tertiary care academic medical center, we present a rare case of a patient from Trinidad, where the skin reactivity to histoplasmin has been reported up to 42% [8], who underwent a two-stage revision due to a *Histoplasma capsulatum* PJI.

## 2. Case Report

**Figure 1:**
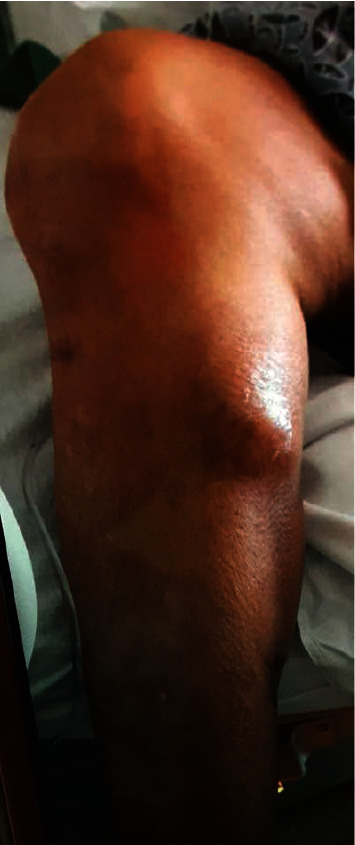
Initial presentation in 2019—large fluid collection distal to the right knee on the medial aspect of calf.

**Figure 2:**
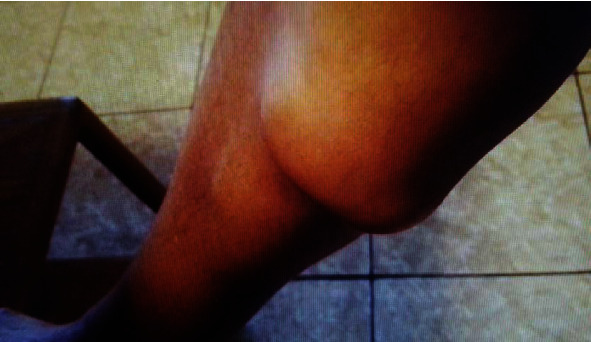
In Trinidad in 2017 before her primary total knee arthroplasty—large collection on the proximal, medial side of her calf distal to the knee.

A 69-year-old female from Trinidad presented to the emergency department at a large tertiary care academic medical center in 2019 with an area of fluctuance over her right proximal calf (Figure 1). The patient described a notable fluid collection on her right lower extremity for several months prior to presentation but experienced an acute episode of pain and increased swelling while visiting her daughter in the United States on vacation that prompted her to seek care. She did not have any medical comorbidities and denied tobacco, alcohol, and drug use. Her past surgical history includes a remote right femur open reduction and internal fixation (ORIF) many years prior and recent primary right total knee arthroplasty (TKA) performed in Trinidad in 2017. She did note that in the weeks prior to her primary right TKA in 2017, she had a similar appearing fluctuance in the same location that spontaneously “popped” (Figure 2) and resolved without treatment.

**Figure 3:**
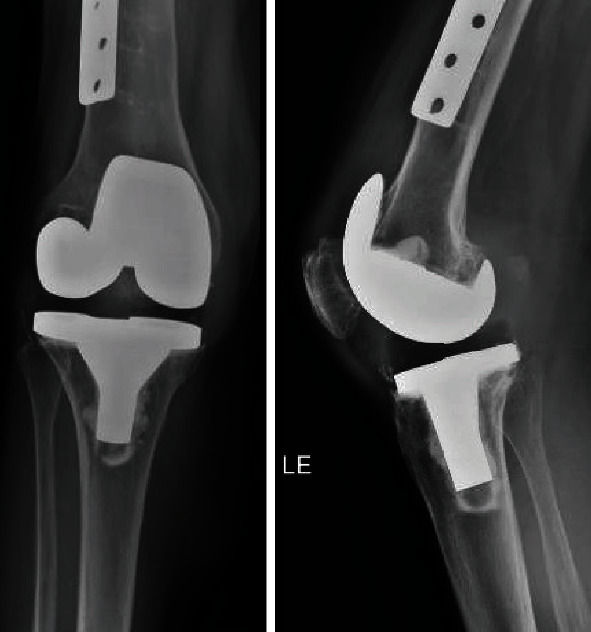
Preoperative total knee prosthesis.

At this visit, she denied any systemic signs of infection but endorsed constant posterior knee pain. Her labs were significant for a white blood cell count (WBC) of 8,000 cells/mm^3^, erythrocyte sedimentation rate (ESR) of 36 mm/hr, and C-reactive protein (CRP) of 1.6 mg/dL. Radiographs were taken and were without fracture, dislocation, or gross signs of infection (Figure 3). Due to the location of the collection, specifically its proximity to the knee joint, a right knee aspiration was performed. The aspirate was sent for gram stain, cell count with differential, and bacterial, acid fast bacilli (AFB), and fungal cultures. A computed tomography (CT) scan with contrast of the right lower extremity was also obtained.

The right lower extremity CT showed a fluid collection measuring 4.2 × 4.1 × 9.7 cm located superior to the medial gastrocnemius muscle. There did not appear to be communication with the joint. The right knee joint aspirate had 45% polys and a total nucleated cell count of 738/mm^3^. In the days following aspiration, no growth was visualized on bacterial, fungal, or AFB cultures. The only positive test was an elevated serum Fungitell level (beta-D-glucan) of 90 pg/mL. Due to the low concern for joint involvement after our initial workup, the patient was brought to the operating room for an incision and drainage of the abscess. Upon incision, the loculation was noted to have “mixed serous and thick cheese-like purulence” that was drained and irrigated.

**Figure 4:**
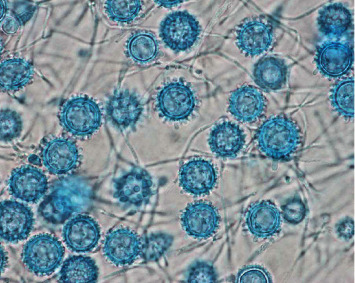
Histoplasma lactophenol cotton blue tape preps, 1000x, from the right knee joint aspirate culture.

**Figure 5:**
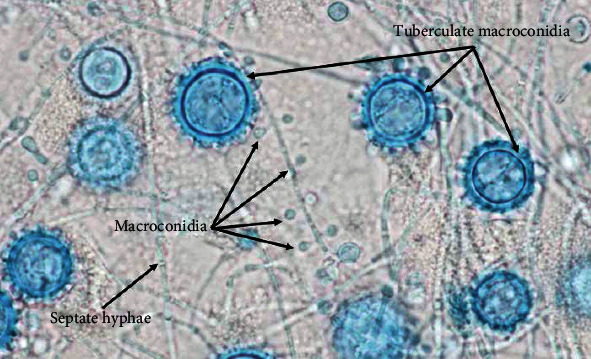
Lactophenol cotton blue tape prep from the right knee joint aspirate culture, 1000x, with labeling of the morphologic structures used for organism identification.

**Figure 6:**
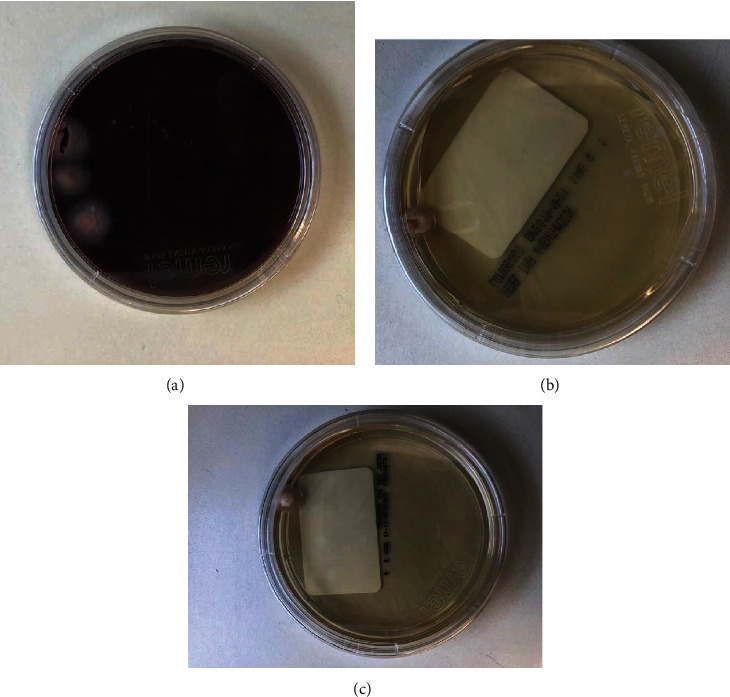
(a) Right knee joint aspirate on BHI agar. (b) Right knee joint aspirate on IMA agar. (c) Right knee joint aspirate on Sabouraud dextrose agar.

After approximately 17 days, the preoperative knee aspirate demonstrated colony growth on brain heart infusion (BHI) agar, inhibitory mold agar (IMA), and Sabouraud dextrose agar, suggesting a fungal etiology. A lactophenol cotton blue tape mount was subsequently used to determine mold morphology for further diagnostic clarification—this preparation demonstrated characteristic findings of *Histoplasma capsulatum* on microscopic analysis, including numerous tuberculate macroconidia and septate hyphae (Figures 4–6). The isolate was also sent to the State Health Lab and has been confirmed to be *H. capsulatum* by nucleic acid hybridization probe. Once diagnosed with a culture positive periprosthetic joint infection, the patient was recommended to undergo a two-stage revision with resection arthroplasty and delayed reimplantation to treat this infection.

In January 2020, the first stage of the revision surgery was performed. The primary TKA and femur hardware was removed. Intraoperative cultures were taken. After a thorough debridement, a nonarticulating spacer was placed. Then, two antibiotic dowel rods using the first batch of cement were prepared. Each dowel was composed of two threaded K-wires coated with this cement. The first batch of cement was composed of 40 mg of polymethyl methacrylate (PMMA) preloaded with gentamicin and subsequently loaded with 100 mg amphotericin B and 200 mg of fluconazole. Methylene blue was added and hand mixed. Once the cement was ready, the K-wires were then coated with this cement and allowed to harden. They were then placed down the femoral and tibial canal, respectively, with appropriate overlap in the joint space.

**Figure 7:**
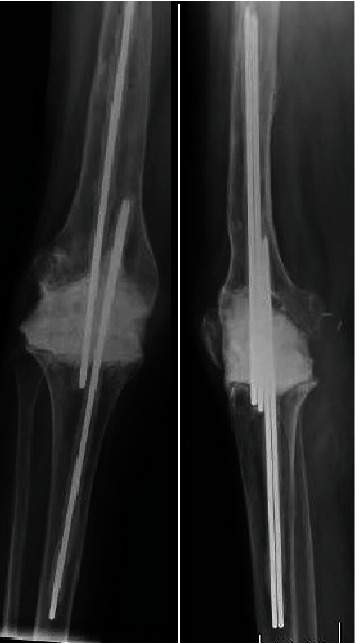
Right knee antibiotic spacer.

After the dowel rods were placed, a second batch of antibiotic cement was prepared for use in the joint space block. This contained 40 mg of PMMA without preloaded antibiotics, 2 grams of cefazolin, 200 mg of amphotericin B, and 200 mg of fluconazole. Fungal PJIs are quite rare, accounting for only 1% of periprosthetic joint infections, and the overwhelming majority of these are secondary to Candida species. The optimal antifungals to be used in a spacer are unknown. We opted to use 2 different antifungals, with different mechanisms of action, in an effort to optimize eradication of this organism. This spacer was used for a short period of time, with partial or no weight bearing on that limb; therefore, mechanical stability was less of a concern. The amount of antifungal used was still well below the recommended 4 grams of antimicrobial per 40 grams of bone cement. After adding cement into the joint space, the leg was held in slight flexion and traction to maintain leg length and ensure a stable postoperative weight-bearing platform. Postoperative X-rays demonstrated satisfactory alignment of the dowels (Figure 7).

Postoperatively, the patient reported severe adverse side effects to intravenous amphotericin B treatment, including dyspnea and severe, diffuse myalgias. The patient received 48 hours of amphotericin B before it was stopped secondary to intolerance. After consultation with the musculoskeletal infectious disease team, her regimen was changed to three months of oral itraconazole for treatment of the *Histoplasma capsulatum* PJI. Her intraoperative cultures also grew *Bacteroides fragilis*, which was treated with 6 weeks of intravenous ertapenem. It was suggested that the presence of a slow-healing open leg wound from the initial abscess debridement was the likely source of this secondary infection.

In July 2020, six months after placement of the joint space block, we proceeded with the second stage of her revision. Repeat joint aspiration was not performed as no effusion was noted at this time. Notable laboratory findings include an ESR and CRP of 16 mm/hr and less than 0.3 mg/dL, respectively.

**Figure 8:**
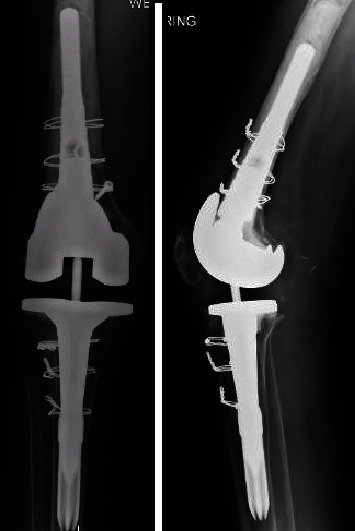
Postop after the second stage of the TKA revision.

During the reimplantation, there were no gross signs of infection, and cultures were taken. We used the Sigma revision system (Depuy TC3 size 2.5 right TC3 femur with size 60 × 15 mm cemented stem with a size 20 cemented femoral sleeve; size 2.5 MBT tibia with a 75 × 16 mm Press-Fit stem; size 15 rotating platform TC3 poly). During the procedure, a tibial tubercle osteotomy was performed due to limited exposure during flexion. Additionally, a fracture of the anterior femoral cortex was noted, requiring cerclage wires and a screw to prevent migration, as seen on X-ray (Figure 8). In the setting of known fungal infection, 400 mg of voriconazole was mixed in the antibiotic cement, and the patient received 6 weeks of oral itraconazole postoperatively. Given the patient's severe adverse reaction to systemic amphotericin B, we opted to avoid using that antifungal medication in the cement used in the two-stage surgery. Intraoperative cultures were held for 14 days and demonstrated no growth.

Recent studies have shown a reduction in the rate of failure after a two-stage revision when patients are given an extended course of pathogen-directed antibiotic therapy. This has yet to be studied in fungal periprosthetic joint infections, and the optimal duration of therapy is unknown. Given the potential morbidity associated with a recurrence of this infection, we discussed the risks and potential benefits of a prolonged course of a systemic antifungal after the second stage, and the patient was in agreement with a 6-week course of oral itraconazole.

At her last follow-up, approximately 7 months after the second stage of the revision, there were no concerns for a recurrent infection.

## 3. Discussion

Periprosthetic joint infections are a devastating complication of total joint arthroplasty that can result in chronic pain, loss of mobility and poor quality of life for the patient, and increased health-care costs resulting from readmissions, additional surgical procedures, and postoperative complications [9]. Identification of the infectious organism is imperative for selection of a specific pharmacologic treatment. In patients with suspected PJI, it is critical to obtain a thorough history that seeks to identify possible risk factors for infection, including delayed wound closure, recent medical and dental procedures, travel history, and animal exposure. Given that the prevalence of skin reactivity to histoplasmin in Trinidad is approximately 42% [8], physicians should include this endemic fungus on their differential as a potential cause of a chronic prosthetic joint infection in this geographic region.

Currently, there are no specific treatment recommendations for a prosthetic joint infection secondary to Histoplasmosis, but we can extrapolate from published guidelines for fungal PJIs in general, that a two-stage exchange is likely indicated [10].

In 2019, Meiyappan et al. [5] published a case report of a Histoplasmosis periprosthetic joint infection that described a different pharmacologic treatment approaches, including the use of an antibiotic spacer cement formulation with 4 grams of vancomycin and 800 mg of amphotericin B. Although this group did not use an antifungal medication in their two-stage reimplantation cement mixture (Simplex P plus tobramycin), the patient has remained infection free at 3.8 years.

Other documented recommendations for cement formulations in fungal infections exist but are not specific to the treatment of Histoplasmosis. In this case, we describe a unique cement formulation that leads to the successful treatment of a patient with *Histoplasma capsulatum* periprosthetic joint infection. This included using antibiotic-coated dowel rods for the static spacer and a cement formulation with amphotericin and fluconazole for the spacer. For the second stage, we recommend using voriconazole mixed into the bone cement. At 7 months post reimplantation, this patient showed no active signs of infection and is progressing well.

Ultimately, in patients with suspected or confirmed periprosthetic joint infection, it is critical to obtain a thorough patient history, to involve an infectious disease specialist team early, and to ensure that the appropriate techniques for two-stage implant revision are employed to optimize patient outcomes.

## Data Availability

The data used in this paper to support the findings of this study are included within the article.
